# Disparities in Breast, Lung, and Cervical Cancer Trials Worldwide

**DOI:** 10.1200/JGO.17.00226

**Published:** 2018-04-11

**Authors:** Ramya Ramaswami, Eduardo Paulino, Adriana Barrichello, Angelica Nogueira-Rodrigues, Alexandra Bukowski, Jessica St. Louis, Paul E. Goss

**Affiliations:** **Ramya Ramaswami**, Imperial College London, Hammersmith Hospital, London, United Kingdom; **Eduardo Paulino**, **Adriana Barrichello**, **Angelica Nogueira-Rodrigues**, **Alexandra Bukowski**, **Jessica St. Louis**, and **Paul E. Goss**, The Global Cancer Institute; and Massachusetts General Hospital, Boston, MA.

## Abstract

**Purpose:**

As cancer burden has risen worldwide, physicians, patients, and their advocates have become aware that the clinical cancer trial research paradigm is not ubiquitous. Furthermore, the number and characteristics of trials that are registered in low- and middle-income countries (LMICs) compared with that in high-income countries (HICs) are unknown.

**Methods:**

We collected retrospective data on trials for breast, lung, and cervical cancer registered in ClinicalTrials.gov or with the WHO International Clinical Trial Registry Platform between 2010 and 2017. The data were then classified as trials within LMICs or HICs using definitions from the World Bank.

**Results:**

Included in these analyses were 6,710 trials, of which 3,164 (47%) were breast cancer trials, 3,283 (49%) were lung cancer trials, and 263 (4%) were cervical cancer trials. There were 1,951 (29%) trials from LMICs and 4,759 (71%) trials from HICs (*P* < .001). Although the proportion of phase III trials in HICs versus LMICs was similar (18% *v* 17%; *P* = .66), the number of phase I trials in LMICs was significantly lower than that of HICs (20% *v* 2%; *P* < .001). For several LMICs with the highest mortality-to-incidence ratios for breast, lung, or cervical cancer, there were no cancer trials registered in the registration data bases searched for this work.

**Conclusion:**

There are differences in access to cancer clinical trials in LMICs compared with HICs. Several factors, such as excessive cost and a lack of infrastructure and expertise, may explain these differences.

## INTRODUCTION

As rates of global cancer incidence and mortality continue to rise, the burden seems to be increasing particularly among low- and middle-income countries (LMICs).^1^ Female breast and lung cancer are the most frequently diagnosed cancers globally, with lung cancer the leading cause of cancer-related death.^2^ Cervical cancer remains a significant burden worldwide, despite screening and vaccination programs.^3^ A proportion of patients with cancer who receive treatment in high-income countries (HICs) may opt to access clinical trials at diagnosis or at any point during their management. These trials commonly investigate the safety and efficacy of new drugs that are in development. Patients who participate in clinical studies are managed with rigorous trial protocols that primarily address the safety of patients and that aim to promptly address toxicities. Establishing research frameworks and institutions also enables better care and can provide more treatment options, which may translate into better outcomes for patients with cancer.^4^

Clinical trials in cancer are a research paradigm that is not applicable globally. The majority of patients with cancer live outside of countries where there is a greater prevalence of cancer clinical trials.^5^ The corresponding paucity of cancer research in the regions of the world with the majority of patients with cancer consequently results in a lack of specific guidance for cancer treatment in these settings. Currently, globally recommended evidence-based treatments do not reflect ethnic, environmental, cultural, or resource differences between HICs and LMICs.^6^ Oncologists in LMICs require specific evidence to create guidelines for the management of their own populations of patients with cancer.^7^ A lack of funding, scarcity of infrastructure linked to cancer research, and the absence of clinical trial regulations or a data-sharing enterprise are some of the main challenges to conducting clinical trial research in LMICs. Small studies have demonstrated that, although there are breast and gynecologic cancer trials in LMICs, as a result of limited research facilities, human resources, and expertise, these trails are insufficient and not necessarily appropriate considering the needs.^8,9^ Clinical trial registries provide information on the type of study conducted, cancer subtype studied, and the progress and completion of ongoing trials. In the current study, we analyze clinical trial registration in LMICs and HICs for breast cancer, lung cancer, and cervical cancer to characterize differences in research efforts for these cancers worldwide.

## METHODS

ClinicalTrials.gov is a trial registration Web site with the largest number of registered studies worldwide.^10^ Registration of these studies represents research priorities and infrastructure in HICs and LMICs. The WHO also maintains the International Clinical Trials Registry Platform to improve access to research for clinicians around the world. Since July 2005, the International Committee of Medical Journal Editors has set the registration of clinical trials as a prerequisite for publication in a high-impact journal^11^; therefore, there is greater impetus among cancer trial researchers to register their studies at the time of patient enrollment. As of 2016, legislation passed in the United States now mandates that the results of studies be reported using existing portals, such as ClinicalTrials.gov.^12^

We searched these trial registries for all breast, lung, and cervical cancer clinical trials between 2010 and 2017 that were registered as phase I, II, or III trials. Patients who were enrolled were older than age 18 years. Any trials that did not include one of these three subtypes of cancer were excluded from additional analysis. Trial registration numbers were compared and any duplicate entries between the data bases were excluded from additional analysis. Countries were divided into HICs and LMICs according to definitions by the World Bank.^13^ For this analysis, if a trial was registered in more than one country, it was counted as a separate entity for each country in which it was registered. Age-standardized incidence and mortality rates per 100,000 cases of breast, cervical, and lung cancers were obtained for countries from GLOBOCAN 2012.^14^ The top 10 cancer mortality-to-incidence (M/I) ratios for HICs and LMICs were tabulated from the total number of cancer trials registered and the total number of metastatic cancers in those countries. We derived trials M/I ratios—M/I ratio multiplied by the number of total breast, lung, or cervical cancer trials for each country—to determine whether these HICs and LMICs developed trials in proportion to their M/I ratios.

Trial characteristics between HICs and LMICs were compared using a two-proportion z-test, with *P* < .05 considered statistically significant. The geographic distribution of registered trials was charted using a Web site tool.^15^

## RESULTS

**Fig A1:**
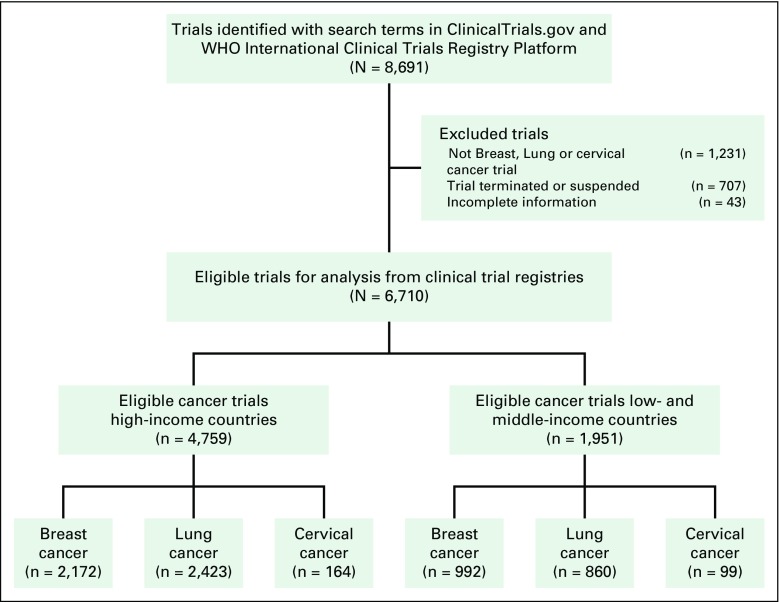
CONSORT diagram. Summary of trials included and excluded in analyses.

A total of 8,691 trials were compiled from both registration data bases, and 6,710 trials were included in the final analysis after excluding those that did not meet our inclusion criteria (Appendix Fig A1). A total of 87 countries had trials registered in the two trial registries. There were 4,759 trials (71%) registered in HICs and 1,951 trials registered in LMICs (29%). Of the selected tumor subtypes, there were 3,283 (49%) lung cancer trials, 3,164 (47%) breast cancer trials, and 263 (4%) cervical cancer trials registered in the stipulated time period. The majority of trials that are included in this analysis were from ClinicalTrials.gov.

### Variation in Trial Registration Among Cancer Subtypes

For all cancer subtypes, fewer than one half of the trials were recruiting patients at the time of data collection. In some cases, registered trials were recorded as being both phase I and II or both phase II and III. If more than one phase was entered, trial protocols were reviewed to confirm the trial phase. Among cervical cancer trials, 84 (32%) were phase I trials, 121 (46%) phase II trials, and 62 (23%) phase III trials. Most breast and lung cancer trials were described as phase II trials. There were similar proportions of phase III trials that were for breast cancer (1,212 trials; 38% of all breast cancer trials), lung cancer (1,013 trials; 30% of all lung cancer trials), and cervical cancer (62 trials; 23% of all cervical cancer trials). A greater proportion of breast cancer trials involved more patients with metastatic cancer (1,009 trials; 32% of all breast cancer trials) compared with lung cancer (589 trials; 18% of all lung cancer trials) and cervical cancer (41 trials; 16% of all cervical cancer trials).

### HICs Versus LMICs

**Fig 1:**
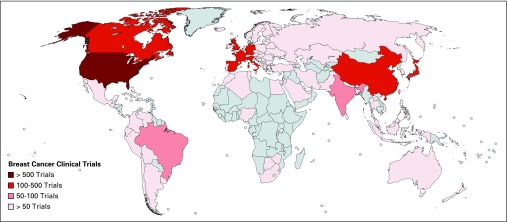
Geographic distribution of clinical trials for breast cancer.

**Fig 2:**
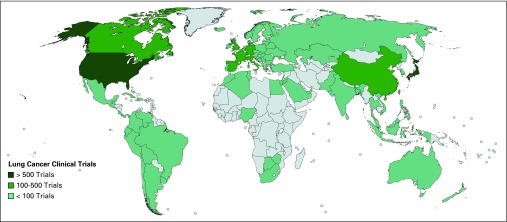
Geographic distribution of clinical trials for lung cancer.

**Fig 3:**
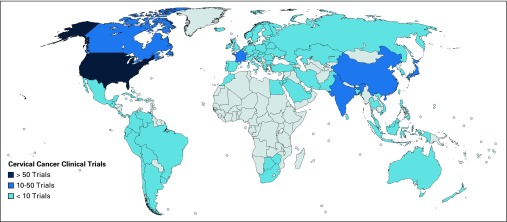
Geographic distribution of clinical trials for cervical cancer.

Median numbers of patients from HICs and LMICs enrolled in breast cancer trials were 129 and 480; for lung cancer trials, 141 and 343 patients; and for cervical cancer trials, 72 and 167 patients, respectively. For all tumor subtypes, there was a higher proportion of phase I trials that originated from HICs versus LMICs (1,361 trials [20% of all studies] *v* 162 trials [2% of all studies]; *P* < .001; Table 1). The proportion of phase III trials was similar between HICs and LMICs (1,120 trials [18% of all eligible studies] *v* 1,167 trials [17% of all eligible studies]; *P* = .66). A greater proportion of the trials that included patients with metastatic lung, breast, or cervical cancers were from HICs compared with LMICs (1,182 trials [18% of all included trials] *v* 457 trials [7% of all included trials]; *P* < .001). There was a similar proportion of trials in HICs and LMICs that investigated biologic therapies (373 trials [6% of all studies] *v* 193 trials [3% of all studies] *P* = .15). There was a statistically significant difference in the proportion of industry-sponsored trials between HICs and LMICs, with evidence of more industry-sponsored trials in HICs (2,833 trials [42% of all studies] *v* 1,520 trials [23% of all studies] *P* < .001). The geographic distribution of studies demonstrated that the United States hosted the largest number of registered trials for breast (Fig 1), lung (Fig 2), and cervical cancer (Fig 3), followed by Canada and countries in Europe. Among LMICs, China and India had a higher number of trials compared with other countries in Asia for breast and cervical cancer (Figs 1 and 3).

**Table 1:**
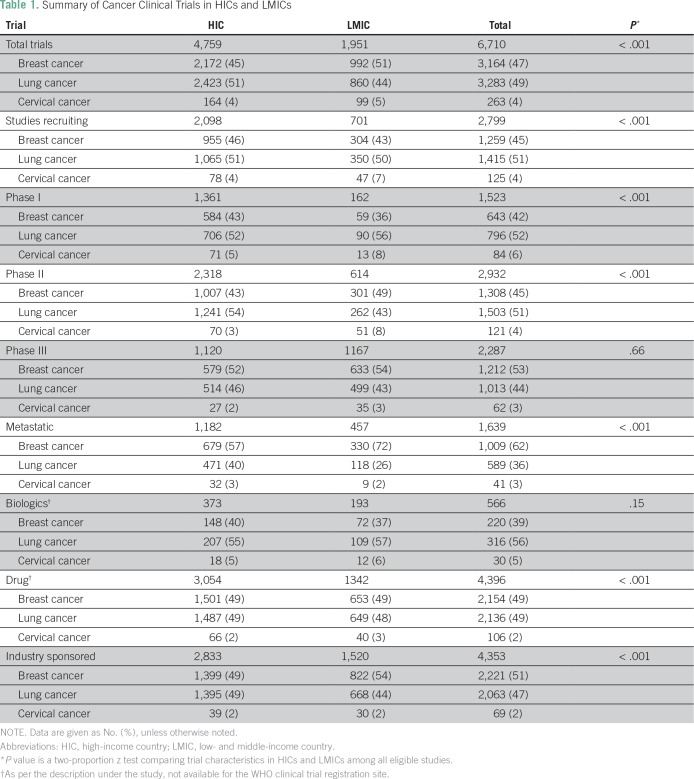
Summary of Cancer Clinical Trials in HICs and LMICs

### Incidence, Mortality, and Trial Distribution

Across breast, cervical, and lung cancers, the respective M/I ratios for these cancers were lower in HICs than in LMICs (Tables 2, 3, and 4). For several LMICs, such as Zimbabwe, Bangladesh, and Haiti, which have the highest breast, lung, or cervical cancer M/I ratios (≥ 0.5), no clinical trials for these cancer subtypes were registered in either registration data base searched. Furthermore, there was a variation in the number of all clinical trials, as such countries as India, South Africa, and Malaysia had a higher number of trials relative to other LMICs.

**Table 2:**
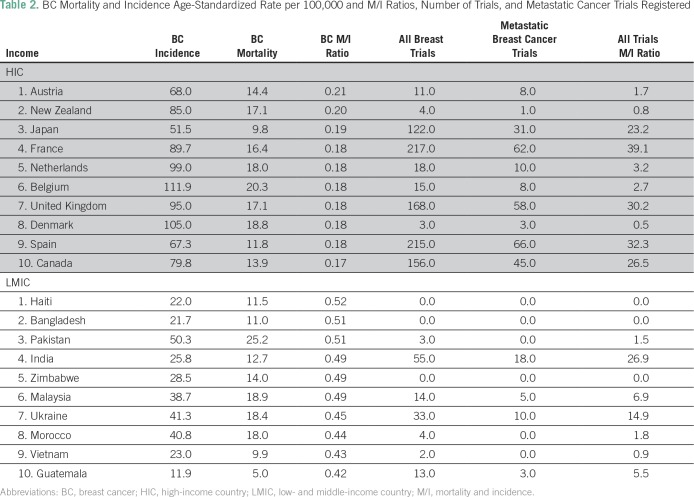
BC Mortality and Incidence Age-Standardized Rate per 100,000 and M/I Ratios, Number of Trials, and Metastatic Cancer Trials Registered

**Table 3:**
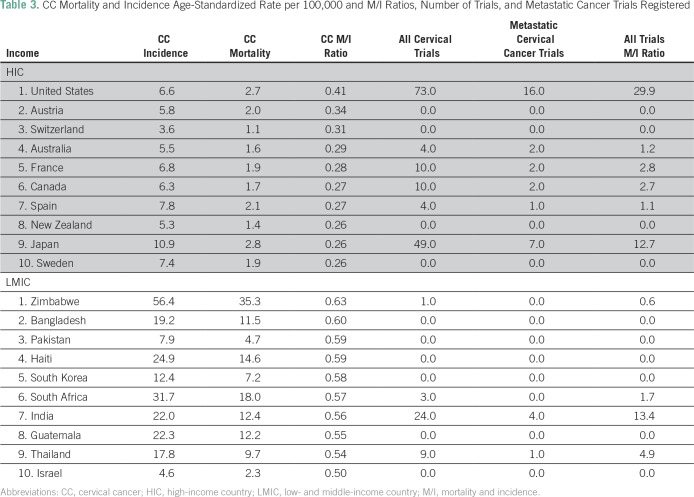
CC Mortality and Incidence Age-Standardized Rate per 100,000 and M/I Ratios, Number of Trials, and Metastatic Cancer Trials Registered

**Table 4:**
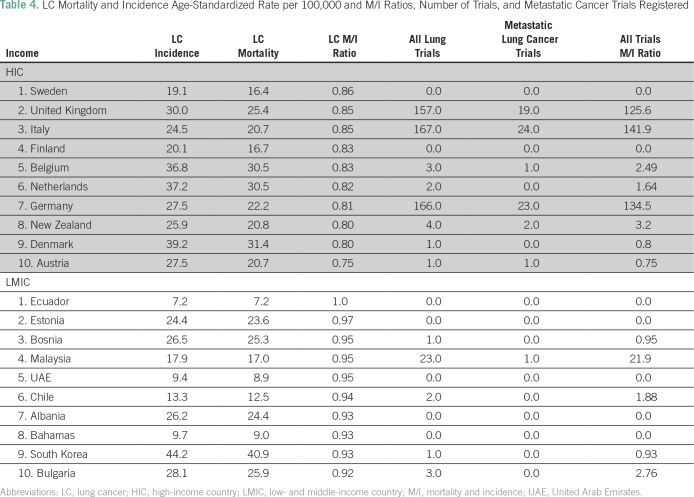
LC Mortality and Incidence Age-Standardized Rate per 100,000 and M/I Ratios, Number of Trials, and Metastatic Cancer Trials Registered

## DISCUSSION

In this analysis of 6,710 breast, lung, and cervical cancer clinical trials registered between 2010 and 2017, it is evident that there are a greater number of registered trials in HICs than in LMICs. Registered trials in HICs were predominantly phase I, industry-sponsored trials that investigated drug therapies. The current study also identified a similar number of phase III trials that were registered in both HICs and LMICs, which implies an increased effort to establish research frameworks in lower-resource regions.

We observed a surprisingly low number of trials registered for cervical cancer over the study time period compared with those for lung and breast in both HICs and LMICs. Industry-sponsored trials represented only 26% of cervical cancer trials compared with more than 60% for breast and lung cancer, despite the fact that multimodality therapies, such as surgery, radiotherapy, and chemotherapy, form the basis of treatment for all three cancer subtypes. The discrepancy in the number of cervical cancer trials versus breast and lung cancer trials may be attributed to the wider use of biologic and targeted therapies for breast and lung cancer, which are often industry sponsored.

Among HICs, a majority of trials for all three tumor subtypes were registered in the United States and countries in Europe. There was an uneven distribution in the number of trials registered throughout LMICs; we found that in some LMICs, such as China, the number of registered trials was nearly equal to that of some HICs, and that many others—for example, countries in Africa—had no trials registered at any point in the specified timeframe. A 2016 study that reviewed registered breast cancer studies in LMICs found that, of the LMICs, Asia had the most trial registrations, with India as the leader in the region.^16^ Similar to the current study, this previously published report demonstrated that few countries on the African continent had clinical trials registered for patients with any of the three cancer subtypes of interest.

Various financial and logistical constraints are recognized obstacles to conducting clinical trials in LMICs, making industry and academic centers less likely to establish clinical trials in LMICs.^17^ Financial barriers are evident when examining both internal and external sources of funding for LMICs. Within countries, many public health systems are already underfunded, and have often proven to be difficult for governments to allocate additional public funding for clinical trials. For example, LMICs account for more than 60% of new cancer cases globally, but represent only 6.2% of global cancer expenditures.^18^ In addition, in 2013, the pharmerging countries of India, China, and Brazil spent 4.0%, 5.6%, and 9.7% of their gross national product, respectively, on health, whereas Japan, Canada, and the United States spent 10.3%, 10.9%, and 17.1%, respectively.^19^ Gaining external funding by participating in industry-sponsored clinical trials could be a potential solution to this problem; however, this solution has its limits as industries that are based in HICs may not prioritize cancer subtypes that are more common in LMICs because of the lower prevalence in their home market and, consequently, the likelihood of decreased revenues. An example of this is observed in the low prevalence of cervical cancer clinical trials globally; 84% of cervical cancer diagnoses and 87% of cervical cancer deaths occur in developing countries,^8^ and there are markedly lower numbers of cervical cancer trials conducted in HICs and LMICs alike compared with other common cancer subtypes.

Logistical barriers to clinical trial administration in LMICs include limited human resources, few and already overburdened health facilities, and uncoordinated research infrastructures within and among countries. The research opportunities afforded to HIC clinicians, who are not hindered by such constraints, differ.^20^ In general, LMICs have far fewer physicians and oncology specialists per capita than the United States, despite a growing burden of disease. Shortages of oncologists and other health professionals limit the ability of a health system to conduct clinical trials. Tertiary cancer centers in LMICs are also often concentrated in large urban centers and are already overburdened by high patient volumes, thus making the additional tasks involved with coordinating research trials difficult.^18,19,21^ In addition, regardless of the country of trial registration, all participating sites need to ensure that there is sufficient rigor in the conduct of a trial and adherence to international standards regarding the care of trial participants. These obstacles could be overcome through global partnership models, such as the Cervix Cancer Research network and the Gynecologic Cancer Intergroup, which encourage trial networks to expand to LMICs, increase long-term collaborations between HICs and LMICs, and help to build local capacity for conducting clinical trials in LMICs.^8,22^

There are many potential benefits of improving clinical trial infrastructure in LMICs, which shoulder a large proportion of the global cancer burden. First, improving the accessibility of HIC-based clinical trials in LMICs and increasing clinical trials that are designed and implemented in LMICs themselves will have immense benefits for global information exchange. Second, the involvement of patients from LMICs in clinical trials will increase the sample size of a study as well as the heterogeneity and generalizability of trials and their results. It would also allow for the creation of evidence-based guidelines and cancer control plans that are relevant to local populations and needs.^8^ Third, some researchers have hypothesized that there is an infrastructure effect, whereby the growth of clinical research infrastructure also acts as a driver of overall health system development by introducing strong organizational and regulatory mechanisms, and building local capacity and expertise in the medical field.^7^

Lastly, increasing access to clinical trials in LMICs would also allow patients access to medicines that might otherwise be inaccessible in resource-limited settings as a result of the financial constraints of both public health systems and individual patients; however, clinical trial recruitment in LMICs may raise ethical concerns regarding patient exploitation. This is an important point to address where clinical trials are led by large pharmaceutical companies. Previous analyses have demonstrated that industry sponsorship, which vigorously registers non-Western trials, may be responsible for the unequal distribution of clinical trials worldwide.^23^ Industry-sponsored studies may recruit more patients in LMICs as a result of the availability of a large number of eager patients who have limited therapeutic options and the absence of regulatory bodies that govern trial conduct in HICs—for example, the US Food and Drug Administration or the European Medicines Agency. Patients in these countries may not have access to these new therapies after the completion of a trial and regulatory approval,^18^ and this is an issue that must be considered when determining whether to open a clinical trial in a low-resource setting or an LMIC. As an example, bevacizumab has been demonstrated to increase survival in women with advanced cervical cancer in a study of 452 women from centers in the United States and Spain.^24^ The prohibitive cost of the bevacizumab regimen precludes its use for the treatment of advanced cervical cancer in LMICs. Cost-effectiveness analyses have demonstrated that, even when the price of bevacizumab is reduced by 75%, the cost per quality-adjusted life month is still three times that of the annual per-capital gross national income of an individual living in an LMIC.^25^ Conversely, bevacizumab has been considered to be the standard treatment of patients with metastatic colon cancer in India, despite evidence from trials that have demonstrated minimal benefit to survival, demonstrating misplaced investment by LMICs in an effort to equalize cancer care.^20,26^ Trial networks and pharmaceutical companies must consider their social and moral obligations to patients with cancer worldwide and ensure free or heavily subsidized access to these drugs after approval.

This analysis has various limitations, most of which stem from the reliability of the data sources. First, ClinicalTrials.gov and the WHO clinical trials registration portals rely on investigators from various countries to use and regularly update their clinical trial entries. Not all investigators will enter trial information in these registries; therefore, the accuracy of information, such as recruitment status, may be limited. Second, we were not able to investigate any change in the trends of trial registration over time, nor were we able to study regional variations within countries. For example, there may be rural areas within the United States or the United Kingdom where limited access to clinical trial facilities may mimic the situation within LMICs.^27^ Third, some trials may have been registered in more than one geographic location, and within this study, they were counted as separate entities to better account for geographic spread; however, this practice may have inflated some trial characteristics, such as the proportion of trials that included patients with metastatic disease. Fourth, as some trials were described as both phase I and II, or both phase II and III, there may be classification errors in describing this particular trial characteristic in the current study. Finally, there is a lack of standardization among different organizations regarding the classification of LMICs and HICs, and use of a classification system that is different from that used in this analysis may yield slightly different results.

In conclusion, this analysis has demonstrated that access to clinical trial registration and implementation in LMICs is limited compared with HICs. The creation and growth of global research collaborations and networks is one mutually beneficial way by which to advance and protect the interests of patients with cancer in LMICs and HICs. Trial conduct and the role of pharmaceutical companies in LMICs requires careful review. Advancing knowledge and understanding of cancer prevention, treatments, and outcomes is a priority for the global cancer community. It is important to increase access to clinical cancer trials in LMICs, strengthen national and regional clinical research infrastructures, and improve knowledge-sharing mechanisms between HICs and LMICs to advance global equity in cancer control.
